# New Viologen-Based Ionic Porous Organic Polymers for Efficient Removal of Anionic Dyes and Hexavalent Chromium (Cr (VI)) from Water

**DOI:** 10.3390/molecules30051123

**Published:** 2025-02-28

**Authors:** Meihan Lu, Lijun Sun, Dongxin Yang, Zewen Nie, Weitao Gong

**Affiliations:** School of Chemical Engineering, Dalian University of Technology, Dalian 116024, China; mhlu0316@mail.dlut.edu.cn (M.L.); 880606ab@mail.dlut.edu.cn (L.S.); ydxdhh@mail.dlut.edu.cn (D.Y.); nzw889900@mail.dlut.edu.cn (Z.N.)

**Keywords:** cationic porous organic polymer, cationic framework, adsorption, dye removal, Cr_2_O_7_^2−^ adsorption

## Abstract

Water pollution is a critical environmental issue in modern society, and adsorption is recognized as a straightforward and efficient water purification technique. In this study, three new viologen-based ionic porous organic polymers were designed and successfully synthesized via a simple approach, and their adsorption properties for water pollutants were evaluated. The cationic nature of these polymers, coupled with their large conjugated π-electron system, physicochemical stability, and aromatic backbone, contributes to their high adsorption capacity and rapid adsorption efficiency for anionic contaminants in water such as Methyl Orange, Congo Red, and Cr (VI). The polymers exhibited maximum adsorption capacities of 1617 mg/g for MO, 3734 mg/g for CR, and 530.22 mg/g for Cr (VI), surpassing most previously reported adsorbents. Furthermore, the polymers maintained a high removal rate even in the presence of competing anions. Effective separation of anionic dyes from mixed solutions could be achieved through simple filtration. These characteristics make them promising candidates for water purification applications.

## 1. Introduction

In recent decades, water pollution has evolved into a critical global issue for human civilization [1,2]. Rapid urbanization and industrialization have led to the introduction of various pollutants into water resources, including toxic organic and inorganic substances such as dyes, pharmaceuticals, heavy metals, microbes, etc. [3,4,5,6]. As a consequence, the effective treatment of these toxic contaminants in wastewater has become a crucial global concern [7]. Researchers are increasingly focused on developing methods to ensure high-quality drinking water devoid of heavy metals, organic dyes, and harmful microorganisms. To tackle this problem, various water remediation techniques have been developed [8], including adsorption [9], coagulation/flocculation [10,11], advanced oxidation [12], photodegradation [13], and membrane separation [14], aiming to remove contaminants and produce pure water effectively. Among the various methods available, adsorption technology has attracted significant attention owing to its cost-effectiveness, operational simplicity, high efficiency, and potential for regeneration [15,16,17]. Consequently, it is of great importance to develop adsorbents that possess rapid adsorption kinetics and substantial capacity. Numerous materials, including zeolite [18], graphene [19], activated carbon [20], mesoporous silica [21], and metal–organic frameworks [22,23,24], have shown remarkable potential for water purification. However, the practical applications of these materials are still restricted because of issues like instability, low removal efficiency, inadequate adsorption capacity, and slow adsorption kinetics.

Porous organic polymers (POPs) are new porous materials distinguished by their extensive pore networks, tunable specific surface areas, excellent chemical and thermal stability, and simple synthesis procedures [25,26,27,28]. Among them, ionic porous organic polymers (iPOPs) represent a novel category of porous materials. Through the introduction of charged groups into the porous network, iPOPs can exhibit functionalities distinct from neutral skeletons [29,30]. These materials possess charged sites that enable electrostatic interactions between ions, thereby enhancing their adsorption capacity [31]. Additionally, the counterions present in iPOPs are exchangeable, which allows for the selection of suitable counterions to effectively modulate the material’s adsorption capacity and selectivity for specific anions [32,33]. Viologen, which contains a bis-cationic 4,4′-bipyridine unit, exists in three different redox states: bis-cationic, radical cationic, and neutral [34,35,36]. Due to its unique electronic structure and multivalent properties, viologen has performed well in many applications. Especially in the development of adsorbent materials, viologen-based porous materials can form strong electrostatic interactions with a wide range of anions due to their cationic structure [37]. To date, various methods have been used to incorporate viologen monomers into porous polymer systems, including the Menshutkin reaction [38], Sonogashira coupling [39], and the Zincke reaction [40,41]. However, these methods often suffer from harsh synthesis conditions, complex procedures, and low yields. On the other hand, most existing studies use isolated viologen in POPs as an adsorbent, with limited research on the adsorption performance of π-extended conjugated viologens in POPs [42]. Therefore, the effect of viologen’s structural variations on adsorption properties requires further investigation.

In this study, we employed a simple synthetic strategy to fabricate viologen-based ionic porous organic polymers. The tetraphenylene vinyl structure, featuring a large conjugated π-electron system and numerous aromatic rings, was selected as the linker. To investigate the impact of structural variations in viologen on adsorption properties, we designed three viologen-based porous organic polymers with different architectures and evaluated their adsorption behaviors. By integrating a benzene ring into the 4,4′-bipyridine structure, we anticipated an improvement in the polymer’s pore structure stability and specific surface area. Moreover, through introducing a methoxy group, an electron-donating substituent on the benzene ring, we intended to enhance the polymer chain rigidity, strengthen intermolecular π–π interactions further, provide additional hydrogen bond donor and acceptor sites, and create multiple mechanisms for interactions with adsorbed molecules. The cationic nature of the polymers, combined with the larger conjugated π-electron system, physicochemical stability, and aromatic backbones, endows them with good water dispersibility and the ability to effectively remove heavy-metal ions and anionic dyes. The adsorption capacities of the three iPOPs for anionic dyes and heavy-metal ions in aqueous solutions were assessed using Methyl Orange (MO) and Congo Red (CR) as target anionic dyes, and hexavalent chromium (Cr (VI)) as the heavy-metal pollutant. The results showed that the polymers exhibited remarkably high adsorption capacities, reaching maximum values of 1617 mg/g for MO, 3734 mg/g for CR, and 530.22 mg/g for Cr (VI), outperforming most previously reported adsorbents. Furthermore, the polymers exhibited a rapid adsorption capacity for heavy-metal ions and anionic dyes, demonstrating the ability to selectively adsorb anionic dyes from binary mixed solutions in just 1 min. Additionally, they facilitated the effective separation of anionic dyes from mixed solutions through simple filtration, highlighting their promising potential as adsorbents.

## 2. Results and Discussion

### 2.1. Preparation of Ionic Porous Organic Polymers

**Scheme 1:**
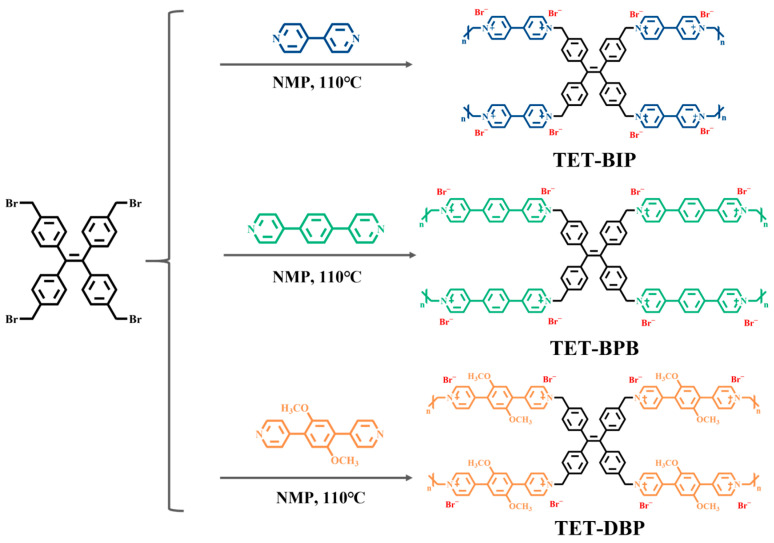
Synthesis routes of three viologen-based iPOPs.

The synthesis procedure for the monomers is described in the Supplementary Materials (Figure S1). The synthetic routes to three iPOPs, TET-BIP, TET-BPB, and TET-DBP, are illustrated in Scheme 1, and they were synthesized through a similar method [43].

### 2.2. Structural Characterization of Materials

**Figure 1:**
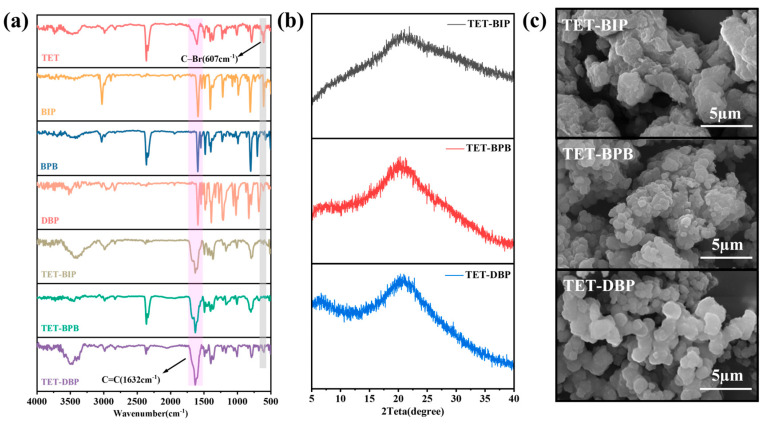
(**a**) FT-IR, (**b**) PXRD, and (**c**) SEM results for TET-BIP, TET-BPB, and TET-DBP.

The structures of the three iPOPs were initially analyzed using infrared spectroscopy, as shown in Figure 1a. In the spectrum of the monomer TET, characteristic absorption peaks at 1632 cm^−1^ correspond to the C=C group, and those at 607 cm^−1^ correspond to the C-Br group. The disappearance of the C-Br band at 607 cm^−1^ and the appearance of the C=C band at 1632 cm^−1^ in the spectra of the three iPOPs confirm the successful polymerization. The thermal stability of the three iPOPs was investigated via heat loss analysis under a nitrogen atmosphere, and these results are shown in Figure S2. TET-BIP began to decompose at approximately 250 °C, with 38% residue remaining at 800 °C. TET-BPB started to decompose at about 300 °C, leaving 30% residue at 800 °C. TET-DBP began to decompose at around 200 °C, with 45% residue remaining at 800 °C. These results indicate that all three iPOPs exhibit good thermal stability. The minor weight loss observed during the initial heating phase is likely attributed to the evaporation of crystalline water and residual solvent molecules confined within the pores. The X-ray diffraction (XRD) patterns of the three iPOPs show broad peaks between 15° and 30° (Figure 1b), confirming their amorphous nature. The scanning electron microscopy (SEM) images, as shown in Figure 1c, reveal that all three iPOPs are amorphous and possess a porous network structure. TET-BIP exhibited irregular sheet-like aggregates with a rough surface, while TET-BPB showed more uniform particles with smaller sizes, relatively regular shapes, and a clear surface structure; TET-DBP displayed a structure similar to TET-BPB but with slightly larger particles that formed chain-like aggregates with inhomogeneous sizes. The N_2_ adsorption–desorption isotherms of the three iPOPs at 77 K were obtained to characterize their specific surface area, porosity, and pore volume (Figure S3). The adsorption isotherms of all iPOPs were similar and classified as type II, indicating the presence of both micro- and mesopores. The specific values are presented in Table S1, showing that all iPOPs exhibit relatively small specific surface areas, which is consistent with their structural properties. TET-BPB demonstrates an increase in pore size and pore volume compared to TET-BIP, likely due to the elongation of the molecular chain. In contrast, TET-DBP exhibits a slightly reduced specific surface area and pore volume compared to TET-BPB. This decrease may be a result of the introduction of methoxy groups on the benzene ring, which can partially block the pores. Several factors contribute to the smaller specific surface areas of these polymers, including the flexible structure of the linker, the charge effects of ionic polymers, and pore blockage by counteracting ions.

### 2.3. Dye Adsorption Performance

The cationic backbone of the iPOPs prompted an investigation into their adsorption properties for pollutants in water. The standard curves of the two dyes (MO, CR) were determined first, and these results are shown in Figure S4. To study adsorption kinetics, 2 mg of TET-BIP, TET-BPB, and TET-DBP was each added to 5 mL of dye solution at room temperature, with the absorbance changes recorded at various time intervals. The UV–Vis spectra showed a significant decrease in absorption at 464 nm for both MO dyes and at 498 nm for both CR dyes over time.

**Figure 2:**
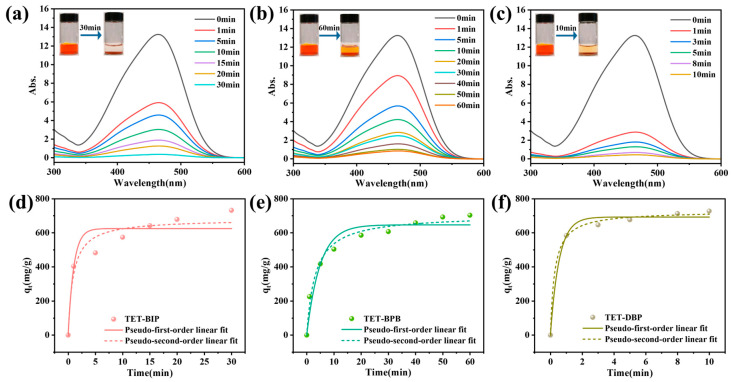
Changes in the UV–Vis spectra of MO solution (300 mg/L) with the addition of (**a**) TET-BIP, (**b**) TET-BPB, and (**c**) TET-DBP (each at 0.4 mg/mL) at different time intervals. The first-order and second-order kinetic models fit the adsorption curves of the MO solution after the addition of (**d**) TET-BIP, (**e**) TET-BPB, and (**f**) TET-DBP.

**Figure 3:**
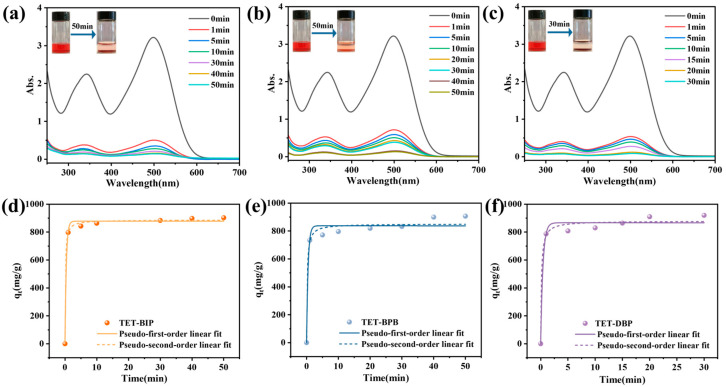
Changes in the UV–Vis spectra of CR solution (400 mg/L) with the addition of (**a**) TET-BIP, (**b**) TET-BPB, and (**c**) TET-DBP (each at 0.4 mg/mL) at different time intervals. The first-order and second-order kinetic models fit the adsorption curves of the CR solution after the addition of (**d**) TET-BIP, (**e**) TET-BPB, and (**f**) TET-DBP.

As shown in Figure 2 and Figure 3, for TET-BIP, approximately 77% of MO was adsorbed within the first 10 min, reaching near equilibrium at around 30 min with a removal efficiency of 97%. For CR, about 91% was adsorbed in the first 10 min, reaching near equilibrium at around 50 min with a removal efficiency of 96%. For TET-BPB, about 68% of MO was adsorbed in the first 10 min, with near complete adsorption at 50 min, resulting in a removal efficiency of 94%. For CR, around 84% was adsorbed in the first 10 min, with near complete adsorption at 50 min, achieving a removal efficiency of 96%. In comparison, TET-DBP exhibited a significantly faster adsorption rate than the other two polymers. About 86% of MO was adsorbed in the first 3 min, reaching near equilibrium at approximately 10 min with a removal efficiency of 97%. For CR, approximately 88% was adsorbed in the first 10 min, with near complete adsorption at around 30 min, resulting in a removal efficiency of 96%. These polymers’ cationic backbone characteristics and porous structures are considered the main driving forces behind their effective adsorption of anionic dyes.

The adsorption processes of MO and CR dyes were analyzed using both a first-order kinetic model (Equation (1)) and a second-order kinetic model (Equation (2)):

(1) $$q_{t}=q_{e}(1-e^{-k_{1}t})$$

(2) $$q_{t}=\frac{k_{2}q_{e}^{2}t}{1+k_{2}q_{e}t}$$

Here, *q_e_* and *q_t_* (mg g^−1^) denote the adsorption capacities of anionic dyes at equilibrium and at time *t* (min), respectively, while *k*_1_ (min^−1^) and *k*_2_ (g mg^−1^ min^−1^) represent the first-order and second-order reaction rate constants, respectively. The parameters derived from the fitting are summarized in Table 1.

The correlation coefficients for the adsorption of MO and CR by TET-BIP, TET-BPB, and TET-DBP are lower when fitted to the first-order kinetic model compared to when fitted to the second-order kinetic model. The second-order model more accurately represents the adsorption behavior of the three iPOPs, indicating that the adsorption of MO and CR primarily follows a chemisorption process. This suggests that the interactions between the polymer backbone and the dye molecules play a dominant role in influencing the adsorption outcomes.

To explore the correlation between dye concentration and adsorption capacity, adsorption isotherm experiments were conducted under a fixed temperature. The adsorption isotherms for the three iPOPs with MO and CR are depicted in Figures S7 and S8. The pertinent parameters were derived utilizing both the Langmuir (Equation (3)) and Freundlich (Equation (4)) isotherm models, as follows:

(3) $$\frac{C_{e}}{q_{e}}=\frac{C_{e}}{q_{m}}+\frac{1}{{(K}_{L}q_{m})}$$

(4) $$lnq_{e}=lnK_{F}+\frac{1}{n}lnC_{e}$$

where *q_m_* represents the theoretical maximum adsorption capacity (mg g^−1^), *q_e_* is the adsorption capacity at various concentrations (mg g^−1^), *C_e_* denotes the equilibrium concentration (mg L^−1^), *K_L_* is the Langmuir isothermal binding constant, and *K_F_* and n are the empirical constants for the Freundlich model.

The Langmuir and Freundlich models were used to fit the *q_e_* versus *C_e_* curves for MO and CR, respectively, on TET-BIP, TET-BPB, and TET-DBP, and the relevant parameters for each model are shown in Table 2. For MO dyes, the R^2^ values of the Freundlich model were higher for all iPOPs, indicating that adsorption followed a multilayer mechanism for each polymer. For CR dyes, the adsorption process on TET-BIP was found to be primarily monolayer adsorption, as indicated by the higher R^2^ value of the Langmuir model compared to the Freundlich model. In contrast, the R^2^ values for the Freundlich model were higher for TET-BPB and TET-DBP, suggesting that the adsorption processes on these polymers were dominated by multimolecular-layer adsorption. The adsorption capacities of TET-BIP for MO and CR were measured at 1617 mg g^−1^ and 3734 mg g^−1^, respectively. In comparison, TET-BPB exhibited adsorption capacities of 1585 mg g^−1^ for MO and 2009 mg g^−1^ for CR, while TET-DBP demonstrated capacities of 1476 mg g^−1^ for MO and 2909 mg g^−1^ for CR. These values surpass the capacities reported for other adsorbents in the literature. As shown in Table 3 and Table 4, the adsorption capacities of all iPOPs are considerably higher than those reported for other adsorbents. Notably, the negative charge density and molecular weight of CR surpass those of MO, leading to a significantly greater adsorption capacity for CR across all iPOPs compared to MO.

**Figure 4:**
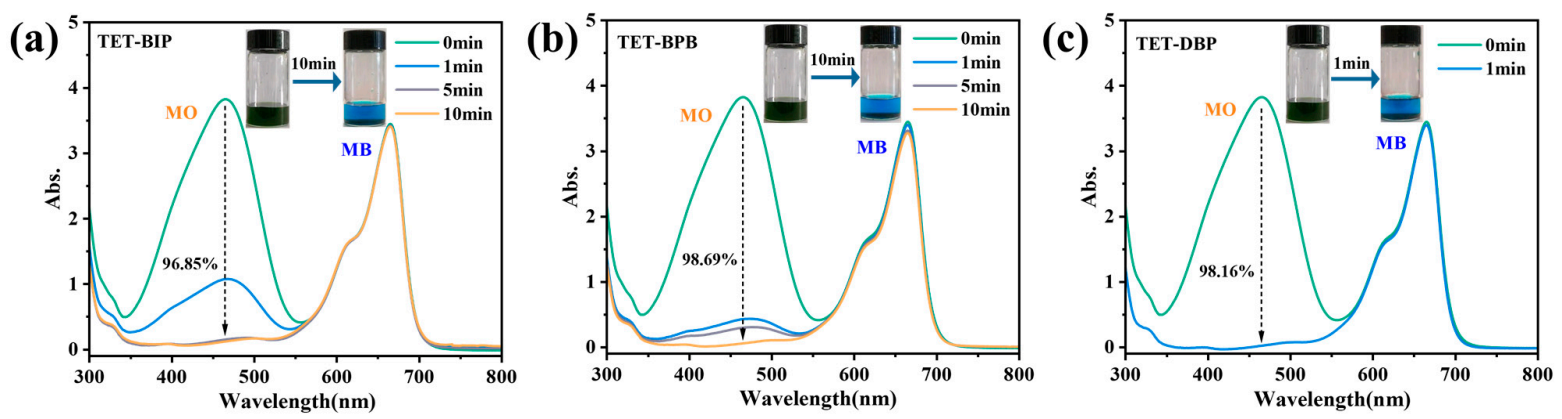
UV–Visible spectral changes in MO and MB binary mixtures upon the addition of (**a**) TET-BIP, (**b**) TET-BPB, and (**c**) TET-DBP.

The separation capacity of dye adsorbents is a critical parameter in the field of dye removal. The viologen-based polymer, characterized by a cationic backbone, demonstrates a high adsorption capacity and removal efficiency for anionic dyes. Consequently, this study investigates its separation selectivity in mixed dye solutions comprising species with varying charges. Specifically, Methyl Orange (MO), an anionic dye, and Methylene Blue (MB), a cationic dye, were selected for this analysis. The adsorption capacity of the three iPOPs for MB dyes was determined first: the iPOPs were added as adsorbents to the MB dye solution (100 mg/L), and their absorption peaks at 665 nm were measured using UV–Vis spectroscopy, showing little to no change (Figure S9). This suggests that the three iPOPs have minimal adsorption capacity for MB. Then, the iPOPs were added to mixed solutions of MO and MB, and the absorption spectra of the filtered solutions over time were recorded. Before adsorption, the UV–Visible spectrum of the mixed MO/MB solution showed characteristic peaks at 462 nm for MO and 665 nm for MB. As shown in Figure 4, all iPOPs rapidly adsorbed MO, with the absorption peak of MO almost disappearing within 10 min. Notably, TET-DBP adsorbed MO almost completely within 1 min, while MB adsorption remained negligible, resulting in the complete separation of the dye molecules. Additionally, the color of the mixed MO/MB solution shifted from dark green to blue. This observation further supports that charge interactions between the polymers and dye molecules play a dominant role in the adsorption process. Consequently, all iPOPs demonstrate high efficiency and rapid adsorption rates in selectively adsorbing anionic dyes from mixtures of anionic and cationic dyes.

**Figure 5:**
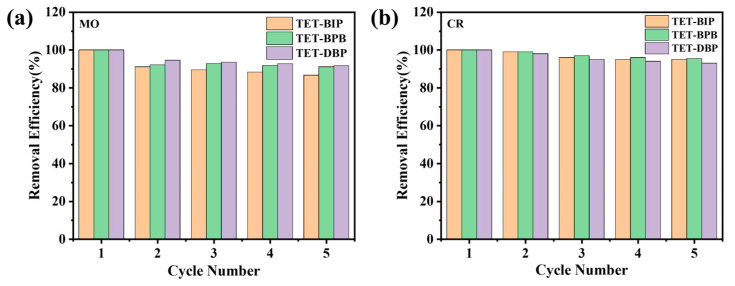
Efficiency of MO (**a**) and CR (**b**) removal by TET-BIP, TET-BPB, and TET-DBP after 5 cycles.

The reusability of the three iPOPs was also evaluated. After washing the polymers with ethanol, methanol, and acetone, the supernatant concentration was measured using a UV–Vis spectrophotometer. Afterward, the adsorbents were reused in a new adsorption–desorption cycle, and the regenerated polymers were tested in subsequent runs. The results show that the iPOPs maintained their trapping capacity after five repetitive cycles, demonstrating efficient reusability (Figure 5).

Based on the experimental results, a potential mechanism for dye adsorption is proposed, with charge interactions identified as the primary factor influencing the adsorption process. The polymer backbone contains nitrogen-positive ions, while bromide ions act as counterions to balance the charge. Consequently, the positively charged viologen-based polymers attract negatively charged dye molecules, allowing the polymers to adsorb anionic dyes through Br–ion exchange [1]. Additionally, the multiple aromatic rings in tetrastyrene enable π–π interactions with the benzene rings of the dye molecules, further enhancing the adsorption capacity [45]. Apart from these essential interactions, the good water dispersibility of the polymers promotes contact between the polymer backbone and the dye molecules, improving the adsorption efficiency. Furthermore, the porous structure of the polymers facilitates the transport and storage of dye molecules. Despite the low specific surface areas of all iPOPs, their high-capacity and rapid adsorption are likely due to efficient adsorption sites and strong chemical interactions. While the benzene ring slightly increases the specific surface area, it may reduce pore connectivity and limit interactions with larger molecules like Congo Red. The addition of methoxy groups does not significantly enhance the specific surface area but appears to improve the adsorption rate and partially restore capacity. These results demonstrate that based on ion exchange principles and electrostatic interactions, the cationic network in these viologen-based polymers enables the rapid and selective adsorption of anionic dyes into their pores.

### 2.4. Cr (VI) Adsorption Performance

**Figure 6:**
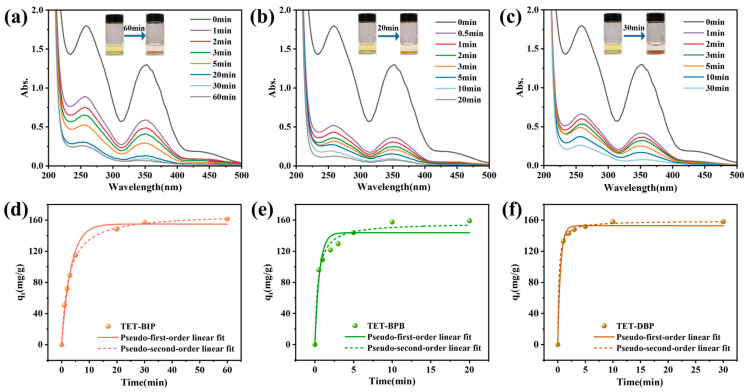
Changes in the UV–Vis spectra of Cr (VI) solution (150mg/L) with the addition of (**a**) TET-BIP, (**b**) TET-BPB, and (**c**) TET-DBP (each at 0.4 mg/mL) at different time intervals. The first-order and second-order kinetic models fit the adsorption curves of the CR solution after the addition of (**d**) TET-BIP, (**e**) TET-BPB, and (**f**) TET-DBP.

Inspired by the adsorption of anionic dyes, we investigated the ability of the three iPOPs to remove Cr (VI) from aqueous solutions. First, the standard curve of Cr_2_O_7_^2−^ was determined, and these results are shown in Figure S10. To investigate the adsorption kinetics, TET-BIP, TET-BPB, and TET-DBP were introduced into an aqueous potassium dichromate solution at room temperature, and the variation in the solution’s absorbance was monitored over time. As shown in Figure 6, for TET-BIP, approximately 78% of Cr (VI) was adsorbed within the first 5 min, and more than 95% was removed from the solution after 60 min. For TET-BPB, around 89% of Cr (VI) was adsorbed in the first 5 min, and more than 94% was removed after 20 min. For TET-DBP, approximately 81% of Cr (VI) was adsorbed within the first 5 min, and more than 94% was removed after 30 min. These results suggest that Cr (VI) rapidly enters the channels of the three iPOPs through ion exchange with Br^−^. The adsorption of Cr (VI) was examined utilizing both first-order and second-order kinetic models. As shown in Table 5, the correlation coefficients for TET-BIP, TET-BPB, and TET-DBP were lower when fitted to the first-order kinetic model compared to the second-order kinetic model. The second-order kinetic model provided a more precise representation of the adsorption behavior of the three iPOPs, suggesting that the adsorption of Cr (VI) by all iPOPs is primarily chemisorptive in nature.

To further assess the adsorption capacity of the three iPOPs for Cr (VI), adsorption isotherm tests were conducted at room temperature. The relevant parameters of the adsorption isotherms were obtained using the Langmuir and Freundlich models (Figure S11). The fitted parameters for each model are presented in Table 6. The data indicate that the adsorption of Cr (VI) by all iPOPs follows the Langmuir isotherm model, suggesting that the adsorption process is characterized by monolayer adsorption. According to the results obtained from the Langmuir model fitting, the maximum adsorption capacities were 468 mg/g for TET-BIP, 530 mg/g for TET-BPB, and 418 mg/g for TET-DBP. These values exceed the capacities reported for other reported adsorbents (Table 7).

**Figure 7:**
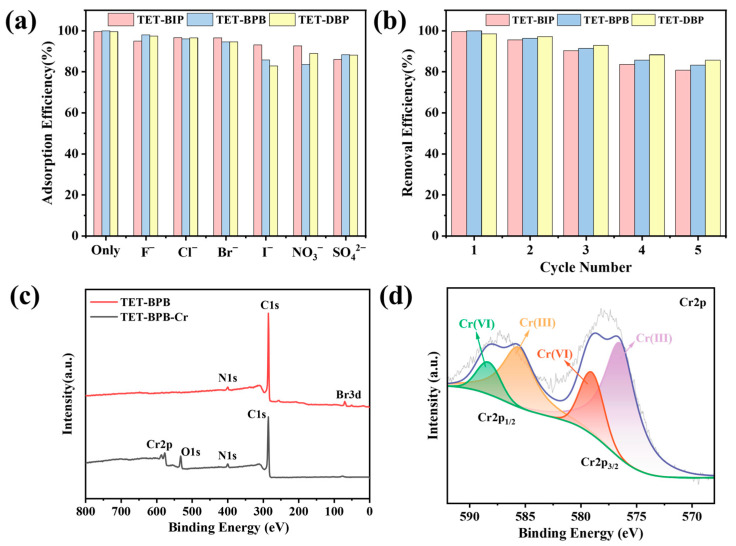
(**a**) Impact of interfering ions on the adsorption capacity of TET-BIP, TET-BPB, and TET-DBP. (**b**) Efficiency of Cr (VI) removal by TET-BIP, TET-BPB, and TET-DBP after 5 cycles. (**c**) XPS spectra of TET-BPB before and after the adsorption of Cr (VI). (**d**) Cr2p XPS peaks of TET-BPB after Cr (VI) adsorption.

Owing to the excellent adsorption capacity and rapid ion exchange properties of the iPOPs, selective adsorption experiments were carried out by adding various interfering anions (including F^−^, Cl^−^, Br^−^, I^−^, NO_3_^−^, and SO_4_^2−^) to the solution. For these experiments, 2 mg of polymer was immersed in 5 mL of K_2_Cr_2_O_7_ solution (100 mg/L), and a twofold excess of the interfering ions was added. The mixture was stirred on a magnetic stirrer for 12 h. As shown in Figure 7a, the addition of F^−^, Cl^−^, and Br^−^ had little effect on the adsorption efficiency, with efficiencies remaining above 95%. In contrast, the addition of I−, NO_3_−, and SO_4_^2−^ affected the adsorption efficiency, likely due to some competition for ion exchange with Cr (VI). However, even in the presence of these interfering ions, the adsorption efficiency of the three iPOPs remained above 80%. These results demonstrate that the iPOPs exhibit highly selective adsorption for Cr (VI).

The reusability of the iPOPs was systematically evaluated. For the desorption experiments, ion exchange was conducted by stirring the polymers overnight in a saturated potassium bromide solution, followed by thorough washing with methanol and ethanol. The concentration of the supernatant was quantified using a UV–Vis spectrophotometer. The results of the repeated regeneration experiments for Cr (VI) adsorption are presented in Figure 7b: all iPOPs demonstrate significant reusability, achieving a removal rate exceeding 99.5% during the initial adsorption cycle. After four cycles, the removal rate remained above 80%, thereby indicating their exceptional regenerative properties.

FTIR and XPS were employed to characterize the polymers after adsorption. The FTIR spectra of the iPOPs, both before and after adsorption, are shown in Figure S12. A characteristic peak at 938 cm^−1^ corresponding to Cr_2_O_7_^2−^ appeared in the infrared spectrum after adsorption, confirming that the polymers adsorbed chromium ions. The XPS spectra of TET-BPB before and after adsorption further support this (Figure 7c), as evidenced by the disappearance of the Br3d peak and the emergence of Cr2p and O1s characteristic peaks. As shown in Figure 7d, the Cr2p spectrum was deconvoluted into four peaks: 576.57 eV (Cr2p_3_/_2_) and 585.76 eV (Cr2p_1_/_2_), attributed to Cr (III), and 579.11 eV (Cr2p_3_/_2_) and 588.48 eV (Cr2p_1_/_2_), attributed to Cr (VI). These results indicate the presence of both Cr (III) and Cr (VI) in the TET-BPB pores. The presence of Cr (III) may be due to the reduction in Cr (VI) during adsorption, where the positively charged nitrogen ions of the polymer provide electrons, lowering the oxidation state of Cr (VI) to Cr (III), and some of which coordinate with the polymer backbone. Ion exchange, electrostatic interactions, and redox reactions are proposed as possible mechanisms for adsorption. The incorporation of a benzene ring allowed the polymer to achieve the highest adsorption capacity for dichromate in monolayer adsorption. However, while methoxy groups enhanced the adsorption rate, they may have reduced the polymer’s electrostatic attraction to dichromate and decreased interactions between nitrogen-positive ions and dichromate, resulting in a relatively lower adsorption capacity. These findings highlight the importance of chemical interactions in designing iPOPs to optimize adsorption properties for specific ions. Even with a modest specific surface area, efficient adsorption can be achieved through strategic functionalization.

## 3. Conclusions

Three viologen-based iPOPs, TET-BIP, TET-BPB, and TET-DBP, were successfully synthesized in this study. The cationic characteristics, extensively conjugated π–electronic systems, physicochemical stability, and aromatic backbones of these polymers significantly enhance their high adsorption capacity and fast adsorption efficiency for anionic dyes, such as MO, CR, and Cr (VI). The maximum adsorption capacities of the polymers, evaluated through Langmuir and Freundlich adsorption isotherms, reached 1617 mg/g for MO, 3734 mg/g for CR, and 530.22 mg/g for Cr (VI), which exceed the performance of most previously reported adsorbents. The adsorption behaviors, analyzed using first-order and second-order kinetic models, suggest that their adsorption of anionic dyes and Cr (VI) is predominantly chemisorptive. In dye adsorption experiments, the polymers exhibited rapid and high-capacity adsorption, as well as the selective extraction of anionic dyes from binary mixtures containing both anionic and cationic dyes, thereby facilitating effective separation. In Cr (VI) adsorption experiments, the polymers demonstrated a high adsorption capacity and satisfactory recoverability in the presence of interfering anions, such as Cl^−^, Br^−^, NO_3_^−^, and SO_4_^2−^, with remarkable selectivity. The proposed mechanisms for adsorption include electrostatic interactions between the cationic backbone and anions, in addition to ion exchange interactions. These findings demonstrate that this cationic porous organic network exhibits dual adsorption capabilities for metal-oxygenated anions and anionic organic dyes, positioning it as a promising adsorbent for the effective removal of pollutants from wastewater. The influence of structural changes on adsorption varies with different substances and involves complex factors, which will be the subject of further investigation in future studies.

## Figures and Tables

**Table 1:** Adsorption kinetics data for TET-BIP, TET-BBP, and TET-DBP towards MO and CR.

Pollutants	Adsorbents	Pseudo-First-Order Model			Pseudo-Second-Order Model		
		*q_e_* (mg g^−1^)	*k*_1_ (min^−1^)	R^2^	*q_e_* (mg g^−1^)	*k*_2_ (g mg^−1^ min^−1^)	R^2^
**MO**	**TET-BIP**	624.7	0.9829	0.902	680.94	0.0016	0.9479
	**TET-BPB**	646.5	0.204	0.9466	705.19	0.0004	0.9826
	**TET-DBP**	692.43	1.8228	0.9903	728.04	0.0051	0.9969
**CR**	**TET-BIP**	877.67	2.3851	0.9962	887	0.0092	0.9981
	**TET-BPB**	837.06	2.0926	0.9757	850.96	0.0063	0.9819
	**TET-DBP**	866.34	2.3899	0.9852	879.49	0.0083	0.9887

**Table 2:** Adsorption isotherm data for TET-BIP, TET-BBP, and TET-DBP towards MO and CR.

Pollutants	Adsorbents	Langmuir Model			Freundlich Model		
		*K_L_*/(L/mg)	*q_m_*/(mg/g)	R^2^	*K_F_*/((mg/g) (L/mg)*^1/n^*)	*n*	R^2^
**MO**	**TET-BIP**	135.94 × 10^−3^	1617	0.958	893.16	9.34	0.9964
	**TET-BPB**	51.61 × 10^−3^	1585	0.9741	640.23	6.56	0.9994
	**TET-DBP**	132.87 × 10^−3^	1476	0.8571	847.7	10.22	0.9993
**CR**	**TET-BIP**	52.33 × 10^−3^	3734	0.9959	1146.44	5.49	0.9779
	**TET-BPB**	13.11 × 10^−3^	2009	0.876	376.29	4.1	0.9973
	**TET-DBP**	19.63 × 10^−3^	2909	0.939	632.73	4.35	0.9977

**Table 3:** Comparison of the maximum adsorption of MO with results published in the literature.

Adsorbents	Description	Adsorption Capacity (mg/g)	Reference
F-PANI2	Porous 3D folic acid–polyaniline hybrid hydrogel	247	[4]
C-NSANaphHCP@Br	Cationic porous organic polymer	1010	[5]
V-CDP	Viologen-based β-cyclodextrin polymer	370	[8]
QUSTiPOP-1	Ionic porous organic polymer	300	[30]
PCP-NH_2_	Hollow amino-rich PCP-NH_2_ microcapsules (inorganic–organic hybrid)	1142	[44]
CMP-Im	Imidazolium–functionalized cationic conjugated microporous polymer	588	[45]
PMSAE−TTDD	Polymeric salicyl active esters	1652	[46]
EA-CDP	β-cyclodextrin-based polymer	602	[47]
{Zn1.5L1}_n_	Metallo-supramolecular polymer	966	[48]
CD-PILs	β-cyclodextrin crosslinked poly(ionic liquid)	1338	[49]
GCA	Composite aerogels prepared from graphene oxide and chitosan	543.4	[50]
MOF-199@AFGO/CS	Metal–organic framework (MOF-199)@aminated graphene oxide aerogel	412	[51]
MoF-525 (Co)	Zr-based porphyrinic MOF	1285.4	[52]
**TET-BIP**	**-**	**1617**	**This work**
**TET-BPB**	**-**	**1585**	**This work**
**TET-DBP**	**-**	**1476**	**This work**

**Table 4:** Comparison of the maximum adsorption of CR with results published in the literature.

Adsorbents	Description	Adsorption Capacity (mg/g)	Reference
QUSTiPOP-1	Ionic porous organic polymer	1074.9	[30]
COP1^++^	Viologen-based covalent organic polymer	928	[38]
CD-PILs	β-cyclodextrin crosslinked poly(ionic liquid)	2058.2	[49]
CDP-DEA	Diethanolamine-modified β-cyclodextrin polymer	813	[53]
CMP-PM-Me	Pyrimidine-based conjugated microporous polymer	400	[54]
Azo-POPs	Azobenzene- and phosphonium-functionalized porous organic polymers	1376.8	[55]
CPAM-Dia/Ce-SA	Cationic polyacrylamide-modified diatomite/Ce(III)–crosslinked sodium alginate hybrid aerogel	1832	[56]
PDA + PEI	Core/shell multi-walled carbon nanotubes/Fe_3_O_4_@polydopamine-polyethyleneimine	1006	[57]
Fe_3_O_4_@Tb/AMP	Magnetic infinite coordination polymer core/shell nanoparticles	909.09	[58]
D-COP	Dithiocarbamate-based covalent organic polymer	1013	[59]
ZnO nanoparticles	ZnO nanoparticles	1554	[60]
Fe_3_O_4_@ZTB-1	MOF-based magnetic material	458	[61]
PEI@MDC	Polyethyleneimine-modified MOF-derived carbon	1723.86	[62]
ZIF-67	Zeolitic imidazolate framework	1044.58	[63]
**TET-BIP**	**-**	**3734**	**This work**
**TET-BPB**	**-**	**2009**	**This work**
**TET-DBP**	**-**	**2909**	**This work**

**Table 5:** Adsorption kinetics data for TET-BIP, TET-BBP, and TET-DBP towards Cr (VI).

Adsorbents	Pseudo-First-Order Model			Pseudo-Second-Order Model		
	*q_e_* (mg g^−1^)	*k*_1_ (min^−1^)	R^2^	*q_e_* (mg g^−1^)	*k*_2_ (g mg^−1^ min^−1^)	R^2^
**TET-BIP**	154.98	0.3003	0.9892	168.77	0.0023	0.9986
**TET-BPB**	143.75	1.6952	0.9372	156.48	0.0159	0.9827
**TET-DBP**	152.78	1.9399	0.9937	158.97	0.0307	0.9995

**Table 6:** Adsorption isotherm data for TET-BIP, TET-BPB, and TET-DBP towards Cr (VI).

Adsorbents	Langmuir Model			Freundlich Model		
	*K_L_*/(L/mg)	*q_m_*/(mg/g)	R^2^	*K_F_*/((mg/g) (L/mg)*^1/n^*)	*n*	R^2^
**TET-BIP**	17.33 × 10^−3^	468	0.9939	67.51	3.25	0.9886
**TET-BPB**	17.74 × 10^−3^	530	0.9989	75.15	3.2	0.9824
**TET-DBP**	20.82 × 10^−3^	418	0.9959	74.64	3.64	0.9881

**Table 7:** Comparison of the maximum adsorption of Cr (VI) with results published in the literature.

Adsorbents	Description	Adsorption Capacity (mg/g)	Reference
CNC-PAH	Poly(acryloyl hydrazide)-grafted cellulose nanocrystal	457.6	[7]
PANI/LDOs	Polyaniline/Mg-Al layered double oxides	409.77	[64]
{[Cu_4_(µ_3_-OH)_2_(mtrb)_2_(1,4-bda)_2_]Br_2_·6H_2_O}_n_	Porous cationic MOF based on a tetranuclear hydroxy-copper(II) cluster	152	[65]
[Cd(tipo)(HCOO)(H_2_O)]·NO_3_·DMF	Microporous cationic MOF	228	[66]
PAN	Polyacrylonitrile/aminated polymeric nanosphere	556	[67]
SFPP	Sulfhydryl-functionalized polymer particles	64.2	[68]
PAMAM	Amine-functionalized carboxylated transition metal carbide composite	318	[69]
ImIP4Br	Imidazolium-based ionic polymer	250	[70]
CMC/PEI	Carboxymethyl cellulose/polyethylenimine hydrogel	312.5	[71]
PAN/P-AC	Polyacrylonitrile/*Pterocladia capillacea*-activated carbon	344.8	[72]
L-Cys/PPY/BT	L–cysteine-doped polypyrrole/bentonite composite	318.5	[73]
**TET-BIP**	-	**468.23**	**This work**
**TET-BPB**	-	**530.22**	**This work**
**TET-DBP**	-	**418.38**	**This work**

## Data Availability

The original contributions presented in this study are included in the article/Supplementary Materials. Further inquiries can be directed to the corresponding author.

## Supplementary Materials

The following supporting information can be downloaded at https://www.mdpi.com/article/10.3390/molecules30051123/s1: Figure S1: Synthesis route of the monomer; Figure S2: Thermogravimetric curves of TET-BIP, TET-BPB, and TET-DBP; Figure S3: N_2_ adsorption–desorption isotherms of (a) TET-BIP, (b) TET-BPB, and (c) TET-DBP (inset: pore size distributions); Figure S4: (a) Standard curve for MO and (b) standard curve for CR; Figure S5: Efficiency of using (a) TET-BIP, (b) TET-BPB, and (c) TET-DBP for MO removal over time; Figure S6: Efficiency of using (a) TET-BIP, (b) TET-BPB, and (c) TET-DBP for CR removal over time; Figure S7: Langmuir and Freundlich model fitting of (a) TET-BIP, (b) TET-BPB, and (c) TET-DBP for MO; Figure S8: Langmuir and Freundlich model fitting of (a) TET-BIP, (b) TET-BPB, and (c) TET-DBP for CR; Figure S9: Changes in the UV–Vis spectra of MB solution with the addition of (a) TET-BIP, (b) TET-BPB, and (c) TET-DBP at different time intervals; Figure S10: Standard curve for Cr_2_O_7_^2−^; Figure S11: Langmuir and Freundlich model fitting of (a) TET-BIP, (b) TET-BPB, and (c) TET-DBP for Cr_2_O_7_^2−^; Figure S12: FTIR spectra of (a) TET-BIP, (b) TET-BPB, and (c) TET-DBP before and after the adsorption of Cr_2_O_7_^2−^; Table S1: BET surface area and porosity parameters of TET-BIP, TET-BPB, and TET-DBP, see [74,75,76].

## References

[1] Fajal S., Hassan A., Mandal W., Shirolkar M.M., Let S., Das N., Ghosh S.K. (2023). Ordered Macro/Microporous Ionic Organic Framework for Efficient Separation of Toxic Pollutants from Water. Angew. Chem. Int. Ed..

[2] Bhowmik K.L., Deb K., Bera A., Debnath A., Saha B. (2018). Interaction of Anionic Dyes with Polyaniline Implanted Cellulose: Organic π-Conjugated Macromolecules in Environmental Applications. J. Mol. Liq..

[3] Liu J., Yu K., Han X., Yu J., Xiang W., Zhao D., Chen B. (2022). Cationic Zr-Based Metal-Organic Framework via Post-Synthetic Alkylation for Selective Adsorption and Separation of Anionic Dyes. Mater. Today Chem..

[4] Das S., Chakraborty P., Ghosh R., Paul S., Mondal S., Panja A., Nandi A.K. (2017). Folic Acid-Polyaniline Hybrid Hydrogel for Adsorption/Reduction of Chromium(VI) and Selective Adsorption of Anionic Dye from Water. ACS Sustain. Chem. Eng..

[5] Shen X., Ma S., Xia H., Shi Z., Mu Y., Liu X. (2018). Cationic Porous Organic Polymers as an Excellent Platform for Highly Efficient Removal of Pollutants from Water. J. Mater. Chem. A.

[6] Samanta P., Chandra P., Dutta S., Desai A.V., Ghosh S.K. (2018). Chemically Stable Ionic Viologen-Organic Network: An Efficient Scavenger of Toxic Oxo-Anions from Water. Chem. Sci..

[7] Park S.-H., Shin S.S., Park C.H., Jeon S., Gwon J., Lee S.-Y., Kim S.-J., Kim H.-J., Lee J.-H. (2020). Poly(Acryloyl Hydrazide)-Grafted Cellulose Nanocrystal Adsorbents with an Excellent Cr(VI) Adsorption Capacity. J. Hazard. Mater..

[8] Li X., Zhou M., Jia J., Jia Q. (2018). A Water-Insoluble Viologen-Based β -Cyclodextrin Polymer for Selective Adsorption toward Anionic Dyes. React. Funct. Polym..

[9] Chakraborty D., Nandi S., Kushwaha R., Kaleeswaran D., Vaidhyanathan R. (2022). Viologen Functionalized C C Bonded Cationic Polymers for Oxo-Anion Pollutant Removal from Aqueous Medium. Mater. Res. Bull..

[10] Kuppusamy S., Thavamani P., Megharaj M., Venkateswarlu K., Lee Y.B., Naidu R. (2016). Potential of Melaleuca Diosmifolia as a Novel, Non-Conventional and Low-Cost Coagulating Adsorbent for Removing Both Cationic and Anionic Dyes. J. Ind. Eng. Chem..

[11] Wang W.-Y., Chiou J.-C., Chen W.-X., Kan C.-W., Lam T.Y.C., Hu H. (2022). Poly(Hexamethylene Biguanide): An Efficient pH-Tolerant and Salt-Intensive Flocculant in the Removal of Anionic Dyes from Wastewater. J. Mater. Sci..

[12] Moreira F.C., Boaventura R.A.R., Brillas E., Vilar V.J.P. (2017). Electrochemical Advanced Oxidation Processes: A Review on Their Application to Synthetic and Real Wastewaters. Appl. Catal. B Environ..

[13] Ajmal A., Majeed I., Malik R.N., Idriss H., Nadeem M.A. (2014). Principles and Mechanisms of Photocatalytic Dye Degradation on TiO2 Based Photocatalysts: A Comparative Overview. RSC Adv..

[14] Li Y., He Y., Zhuang J., Shi H. (2022). Hierarchical Microsphere Encapsulated in Graphene Oxide Composite for Durable Synergetic Membrane Separation and Fenton-like Degradation. Chem. Eng. J..

[15] Skorjanc T., Shetty D., Gándara F., Ali L., Raya J., Das G., Olson M.A., Trabolsi A. (2020). Remarkably Efficient Removal of Toxic Bromate from Drinking Water with a Porphyrin–Viologen Covalent Organic Framework. Chem. Sci..

[16] Yu S.-B., Lyu H., Tian J., Wang H., Zhang D.-W., Liu Y., Li Z.-T. (2016). A Polycationic Covalent Organic Framework: A Robust Adsorbent for Anionic Dye Pollutants. Polym. Chem..

[17] Li M., Zhao H., Lu Z.-Y. (2020). Highly Efficient, Reversible Iodine Capture and Exceptional Uptake of Amines in Viologen-Based Porous Organic Polymers. RSC Adv..

[18] Zheng J., Lan D., Zhang S., Wei F., Liu T., Gao Z., Wu G. (2025). Zeolite Imidazolate Framework Derived Efficient Absorbers: From Morphology Modulation to Component Regulation. J. Alloys Compd..

[19] Li Y., Liu H., Nie R., Li Y., Li Q., Lei Y., Guo M., Zhang Y. (2024). Highly Efficient Adsorption of Anionic Dyes on a Porous Graphene Oxide Nanosheets/Chitosan Composite Aerogel. Ind. Crops Prod..

[20] Li J., Wang S., Peng J., Lin G., Hu T., Zhang L. (2018). Selective Adsorption of Anionic Dye from Solutions by Modified Activated Carbon. Arab. J. Sci. Eng..

[21] Akpotu S.O., Moodley B. (2018). Effect of Synthesis Conditions on the Morphology of Mesoporous Silica from Elephant Grass and Its Application in the Adsorption of Cationic and Anionic Dyes. J. Environ. Chem. Eng..

[22] Zhang Q., Yu J., Cai J., Zhang L., Cui Y., Yang Y., Chen B., Qian G. (2015). A Porous Zr-Cluster-Based Cationic Metal–Organic Framework for Highly Efficient Cr_2_O_7_^2−^ Removal from Water. Chem. Commun..

[23] Liu S.-N., Wang H.-N., Feng M.-L., Yang K.-L., Cai S., Fan J., Zhang W.-G., Zheng S.-R. (2023). Metal–Organic Cage Extended Amorphous Network via Anionic Organic Linkers for Cr_2_O_7_^2−^ and Iodine Adsorption on Nanopores. ACS Appl. Nano Mater..

[24] Desai A.V., Manna B., Karmakar A., Sahu A., Ghosh S.K. (2016). A Water-Stable Cationic Metal–Organic Framework as a Dual Adsorbent of Oxoanion Pollutants. Angew. Chem. Int. Ed..

[25] He X., Zhang S.-Y., Tang X., Xiong S., Ai C., Chen D., Tang J., Pan C., Yu G. (2019). Exploration of 1D Channels in Stable and High-Surface-Area Covalent Triazine Polymers for Effective Iodine Removal. Chem. Eng. J..

[26] Škorjanc T., Shetty D., Olson M.A., Trabolsi A. (2019). Design Strategies and Redox-Dependent Applications of Insoluble Viologen-Based Covalent Organic Polymers. ACS Appl. Mater. Interfaces.

[27] Sarkar S., Chakraborty A., Nag P., Singh S., Munjal R., Vennapusa S.R., Jha H.C., Mukhopadhyay S. (2024). Role of Charge Density and Surface Area of Tailored Ionic Porous Organic Polymers for Adsorption and Antibacterial Actions. ACS Appl. Mater. Interfaces.

[28] Qu W., Zhang S., Dong K., Deng X., Gong W., Ning G. (2021). Construction of Rigid Ionic Porous Organic Polymers (iPOPs) via Zincke Reaction with Tunable Absorption Behaviors. J. Porous Mater..

[29] Liu M., Xu Q., Zeng G. (2024). Ionic Covalent Organic Frameworks in Adsorption and Catalysis. Angew. Chem. Int. Ed..

[30] Jiao S., Deng L., Zhang X., Zhang Y., Liu K., Li S., Wang L., Ma D. (2021). Evaluation of an Ionic Porous Organic Polymer for Water Remediation. ACS Appl. Mater. Interfaces.

[31] Jhariat P., Panda T. (2024). Recent Advancement in Viologen Functionalized Porous Organic Polymers (vPOPs) for Energy and Environmental Remediation. Mater. Adv..

[32] Zadehnazari A., Khosropour A., Zarei A., Khazdooz L., Amirjalayer S., Auras F., Abbaspourrad A. (2024). Viologen-Derived Covalent Organic Frameworks: Advancing PFAS Removal Technology with High Adsorption Capacity. Small.

[33] Zhou X.-H., Fan Y., Li W.-X., Zhang X., Liang R.-R., Lin F., Zhan T.-G., Cui J., Liu L.-J., Zhao X. (2020). Viologen Derivatives with Extended π-Conjugation Structures: From Supra-/Molecular Building Blocks to Organic Porous Materials. Chin. Chem. Lett..

[34] Sarkar S., Ghosh T., Chakrobarty A., Majhi J., Nag P., Bandyopadhyay A., Vennapusa S.R., Kumar R., Mukhopadhyay S. (2023). Exploring a Redox-Active Ionic Porous Organic Polymer in Environmental Remediation and Electrochromic Application. ACS Appl. Mater. Interfaces.

[35] Li G., Song R., Ma W., Liu X., Li Y., Rao B., He G. (2020). π-Extended Chalcogenoviologens with Stable Radical State Enable Enhanced Visible-Light-Driven Hydrogen Evolution and Static/Dynamic Electrochromic Displays. J. Mater. Chem. A.

[36] Tan T., He S., He G., Chen J. (2023). A Stimuli-Responsive Viologen-Containing Polymer for Use in Electrochromic Devices and Amine-Detecting Paper. New J. Chem..

[37] Skorjanc T., Shetty D., Gándara F., Pascal S., Naleem N., Abubakar S., Ali L., Mohammed A.K., Raya J., Kirmizialtin S. (2022). Covalent Organic Framework Based on Azacalix[4]Arene for the Efficient Capture of Dialysis Waste Products. ACS Appl. Mater. Interfaces.

[38] Das G., Skorjanc T., Prakasam T., Nuryyeva S., Olsen J.-C., Trabolsi A. (2017). Microwave-Assisted Synthesis of a Viologen-Based Covalent Organic Polymer with Redox-Tunable Polarity for Dye Adsorption. RSC Adv..

[39] Akhtaruzzaman M., Tomura M., Nishida J., Yamashita Y. (2003). Linear Molecules with Ethynylpyridine and Bisbenzothiadiazole Units. Synth. Met..

[40] Das G., Skorjanc T., Sharma S.K., Gándara F., Lusi M., Shankar Rao D.S., Vimala S., Krishna Prasad S., Raya J., Han D.S. (2017). Viologen-Based Conjugated Covalent Organic Networks via Zincke Reaction. J. Am. Chem. Soc..

[41] Chen G., Huang X., Zhang Y., Sun M., Shen J., Huang R., Tong M., Long Z., Wang X. (2018). Constructing POSS and Viologen-Linked Porous Cationic Frameworks Induced by the Zincke Reaction for Efficient CO_2_ Capture and Conversion. Chem. Commun..

[42] Liu L., Qu W.-D., Dong K.-X., Qi Y., Gong W.-T., Ning G.-L., Cui J.-N. (2021). An Anthracene Extended Viologen-Incorporated Ionic Porous Organic Polymer for Efficient Aerobic Photocatalysis and Antibacterial Activity. Chem. Commun..

[43] Tang J., Yu S., Liu C., Wang H., Zhang D., Li Z. (2019). A Highly Stable Porous Viologen Polymer for the Catalysis of Debromination Coupling of Benzyl Bromides with High Recyclability. Asian J. Org. Chem..

[44] Fu J., Zhu J., Wang Z., Wang Y., Wang S., Yan R., Xu Q. (2019). Highly-Efficient and Selective Adsorption of Anionic Dyes onto Hollow Polymer Microcapsules Having a High Surface-Density of Amino Groups: Isotherms, Kinetics, Thermodynamics and Mechanism. J. Colloid Interface Sci..

[45] Wang S., Meng X., Luo H., Yao L., Song X., Liang Z. (2022). Post-Synthetic Modification of Conjugated Microporous Polymer with Imidazolium for Highly Efficient Anionic Dyes Removal from Water. Sep. Purif. Technol..

[46] Zhang X., Li Z., Lin S., Théato P. (2020). Fibrous Materials Based on Polymeric Salicyl Active Esters as Efficient Adsorbents for Selective Removal of Anionic Dye. ACS Appl. Mater. Interfaces.

[47] Jiang H.-L., Xu M.-Y., Xie Z.-W., Hai W., Xie X.-L., He F.-A. (2020). Selective Adsorption of Anionic Dyes from Aqueous Solution by a Novel β-Cyclodextrin-Based Polymer. J. Mol. Struct..

[48] Jena S.R., Mandal T., Choudhury J. (2022). 3D Metallo-Supramolecular Polymer for Fast and Efficient Removal of Anionic Dyes from Water. ACS Appl. Polym. Mater..

[49] Chen J., Bao C., Chen M., Wang Y., Xu X., Yang T., Shang C., Zhang Q. (2023). β-Cyclodextrin-Scaffolded Crosslinked Poly(Ionic Liquid)s for Ultrafast Removal of Multiple Pollutants: Insight into Adsorption Performance and Mechanism. Chem. Eng. J..

[50] Shi Y., Song G., Li A., Wang J., Wang H., Sun Y., Ding G. (2022). Graphene Oxide-Chitosan Composite Aerogel for Adsorption of Methyl Orange and Methylene Blue: Effect of pH in Single and Binary Systems. Colloids Surf. Physicochem. Eng. Asp..

[51] Zhang W., Huang T., Ren Y., Wang Y., Yu R., Wang J., Tu Q. (2021). Preparation of Chitosan Crosslinked with Metal-Organic Framework (MOF-199)@aminated Graphene Oxide Aerogel for the Adsorption of Formaldehyde Gas and Methyl Orange. Int. J. Biol. Macromol..

[52] Lu H., Yang Q., Huang B., Qi J., Wang R., Zhou Q., Chen Q., Zhu L., Jin J., Kong Y. (2023). Removal Performance and Adsorption Kinetics of Dyes by a Co-Based Metal Organic Framework. Microporous Mesoporous Mater..

[53] Xu M.-Y., Jiang H.-L., Xie Z.-W., Li Z.-T., Xu D., He F.-A. (2020). Highly Efficient Selective Adsorption of Anionic Dyes by Modified β-Cyclodextrin Polymers. J. Taiwan Inst. Chem. Eng..

[54] Liu Y., Cui Y., Zhang C., Du J., Wang S., Bai Y., Liang Z., Song X. (2018). Post-Cationic Modification of a Pyrimidine-Based Conjugated Microporous Polymer for Enhancing the Removal Performance of Anionic Dyes in Water. Chem. Eur. J..

[55] Cheng Y., Chen Y., He M., Zhou N., Meng X., Dai Z., Xiong Y. (2024). Photo-Tunable Ultrafast Removal of Organic Dyes by Azobenzene and Phosphonium Functionalized Porous Organic Polymers. Sep. Purif. Technol..

[56] Li B., Zhao Y. (2023). Facile Synthesis and Ultrastrong Adsorption of a Novel Polyacrylamide-Modified Diatomite/Cerium Alginate Hybrid Aerogel for Anionic Dyes from Aqueous Environment. Int. J. Biol. Macromol..

[57] Zhao S., Zhan Y., Wan X., He S., Yang X., Hu J., Zhang G. (2020). Selective and Efficient Adsorption of Anionic Dyes by Core/Shell Magnetic MWCNTs Nano-Hybrid Constructed through Facial Polydopamine Tailored Graft Polymerization: Insight of Adsorption Mechanism, Kinetic, Isotherm and Thermodynamic Study. J. Mol. Liq..

[58] Huang W., Xu J., Lu D., Deng J., Shi G., Zhou T. (2018). Rational Design of Magnetic Infinite Coordination Polymer Core-Shell Nanoparticles as Recyclable Adsorbents for Selective Removal of Anionic Dyes from Colored Wastewater. Appl. Surf. Sci..

[59] Thurakkal L., Porel M. (2023). Removal of Cationic and Anionic Dyes from Waste Water in Five Seconds by a Facile Synthesis of a Covalent Organic Polymer. Eur. Polym. J..

[60] Zhang F., Chen X., Wu F., Ji Y. (2016). High Adsorption Capability and Selectivity of ZnO Nanoparticles for Dye Removal. Colloids Surf. Physicochem. Eng. Asp..

[61] Han L.-J., Ge F.-Y., Sun G.-H., Gao X.-J., Zheng H.-G. (2019). Effective Adsorption of Congo Red by a MOF-Based Magnetic Material. Dalton Trans..

[62] Hu N., Yu J., Hou L., Shi C., Li K., Hang F., Xie C. (2023). Amine-Functionalized MOF-Derived Carbon Materials for Efficient Removal of Congo Red Dye from Aqueous Solutions: Simulation and Adsorption Studies. RSC Adv..

[63] Guo X., Kong L., Ruan Y., Diao Z., Shih K., Su M., Hou L., Chen D. (2020). Green and Facile Synthesis of Cobalt-Based Metal–Organic Frameworks for the Efficient Removal of Congo Red from Aqueous Solution. J. Colloid Interface Sci..

[64] Long F.-L., Niu C.-G., Tang N., Guo H., Li Z.-W., Yang Y.-Y., Lin L.-S. (2021). Highly Efficient Removal of Hexavalent Chromium from Aqueous Solution by Calcined Mg/Al-Layered Double Hydroxides/Polyaniline Composites. Chem. Eng. J..

[65] Lv X.-X., Shi L.-L., Li K., Li B.-L., Li H.-Y. (2017). An Unusual Porous Cationic Metal–Organic Framework Based on a Tetranuclear Hydroxyl-Copper(II) Cluster for Fast and Highly Efficient Dichromate Trapping through a Single-Crystal to Single-Crystal Process. Chem. Commun..

[66] Guo M., Guo H., Liu S., Sun Y., Guo X. (2017). A Microporous Cationic Metal–Organic Framework for the Efficient Removal of Dichromate and the Selective Adsorption of Dyes from Water. RSC Adv..

[67] Qi J., Zeng M., Zhu Z., Zhou Y., Sun X., Li J. (2022). Polyacrylonitrile/Aminated Polymeric Nanosphere Nanofibers as Efficient Adsorbents for Cr(VI) Removal. Molecules.

[68] Wang Y., Lu W., Jing F., Jin M., He X., Xue Y., Hu Y., Yu R. (2024). Sulfhydryl-Functionalized Amino Group Polymer Microcomposites toward Efficient Adsorption of Aqueous Cr(VI), As(III), Cd(II), and Pb(II). Langmuir.

[69] Geng L., Xu W., Chang S., Chen S. (2024). Polyamidoamine Dendrimers Modified MXene and Its High-Performance Adsorption Behavior for Cr(VI). J. Mol. Liq..

[70] Zhang Y., Zhong H., Lin Z. (2021). Quaternary Amine Synthesized Ionic Polymer for Efficient Removal of Cr(VI) in Waste Water. Surf. Interfaces.

[71] Song L., Liu F., Zhu C., Li A. (2019). Facile One-Step Fabrication of Carboxymethyl Cellulose Based Hydrogel for Highly Efficient Removal of Cr(VI) under Mild Acidic Condition. Chem. Eng. J..

[72] Shoaib A.G.M., El Nemr A., El Sikaily A., Masoud M.S., Ramadan M.S. (2022). Amidoxime Modification of Polyacrylonitrile/*Pterocladia capillacea*-Derived Activated Carbon Composite for Adsorption of Toxic Chromium from Aquatic Environment. Carbon Lett..

[73] Wang L., Muhammad H., Laipan M., Fan X., Guo J., Li Y. (2022). Enhanced Removal of Cr(VI) and Mo(VI) from Polluted Water Using L-Cysteine Doped Polypyrrole/Bentonite Composite. Appl. Clay Sci..

[74] Zhu Y.-X., Wei Z.-W., Pan M., Wang H.-P., Zhang J.-Y., Su C.-Y. (2016). A New TPE-Based Tetrapodal Ligand and Its Ln(III) Complexes: Multi-Stimuli Responsive AIE (Aggregation-Induced Emission)/ILCT(Intraligand Charge Transfer)-Bifunctional Photoluminescence and NIR Emission Sensitization. Dalton Trans..

[75] Scott H.S., Shivanna M., Bajpai A., Madden D.G., Chen K.-J., Pham T., Forrest K.A., Hogan A., Space B., Perry Iv J.J. (2017). Highly Selective Separation of C_2_ H_2_ from CO_2_ by a New Dichromate-Based Hybrid Ultramicroporous Material. ACS Appl. Mater. Interfaces.

[76] Feng Y., Das P.J., Young R.M., Brown P.J., Hornick J.E., Weber J.A., Seale J.S.W., Stern C.L., Wasielewski M.R., Stoddart J.F. (2022). Alkoxy-Substituted Quadrupolar Fluorescent Dyes. J. Am. Chem. Soc..

