# Supplementary material for: New Viologen-Based Ionic Porous Organic Polymers for Efficient Removal of Anionic Dyes and Hexavalent Chromium (Cr (VI)) from Water
Source: Molecules. 2025 Feb 28;30(5):1123. doi: 10.3390/molecules30051123 (PMC11901743; doi:10.3390/molecules30051123)
Supplement: Supplementary file 1 [file molecules-30-01123-s001.zip › molecules-3495840-supplementary.pdf]

Supplementary Materials

New Viologen-Based Ionic Porous Organic Polymers for Efficient Removal of Anionic Dyes and Hexavalent Chromium (Cr (VI)) from Water

Meihan Lu, Lijun Sun, Dongxin Yang, Zewen Nie, Weitao Gong*

School of Chemical Engineering, Dalian University of Technology,

Dalian 116024, China

* Correspondence: *wtgong@dlut.edu.cn*

Materials and Methods

1. Materials characterizations

All the commercially available materials and reagents were obtained from suppliers and used without further purification except when specified. The FT-IR spectra of the polymers were tested on a JASCO IR-4100 spectrometer using the conventional KBr plate method in the range of 400-4000 cm^{-1}. 1H NMR spectra were recorded on a Bruker Avance II 400 NMR instrument. Powder X-ray diffraction (PXRD) in the range of 5°~40° was measured on a Bruker D8 Advance type diffractometer (40 kV, 200 mA) at a scan rate of 10°/min. Thermogravimetric analysis (TGA) of the samples was carried out using a Mettler Toledo TGA/DSC3+ thermal analyser under a flowing nitrogen atmosphere, and the samples were heated from room temperature to 800 °C at a ramp rate of 10°/min. SEM patterns were recorded using a FEI Nova NanoSEM 450 scanning electron microscope with an operating voltage of 20 KV. The specific surface area, nitrogen adsorption and desorption isotherms (77 K), and pore size distribution (298.15 K) of the polymers were collected using a Quantachrome 2QDS-MP-30 surface area and porosity analyser. Before the adsorption analysis, the samples were pre-treated and degassed under vacuum at 120 °C for 12 h, with air conditioning to control the room temperature to control the 298 K. The adsorption properties of all samples were tested using JASCO V750 UV-Vis spectrophotometer.

2. Experimental Sections

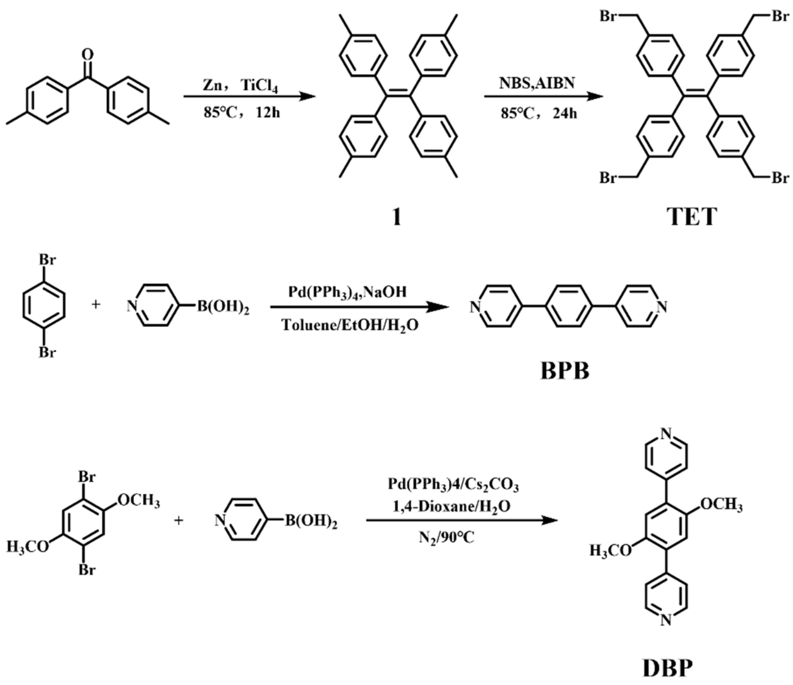

Figure S1. Synthesis route of the monomer.

Synthesis of TET.

Synthesis of 1,1,2,2-tetra-kis(4-(bromomethyl)phenyl)-ethene(TET) refers to previous literature (**Figure S1**) [1].

1,1,2,2-Tetra-p-tolylethylene (1): Zinc powder (3.0 g, 45.9 mmol) was added to a 250 mL triple-necked flask under an argon atmosphere, followed by 75 mL of dry tetrahydrofuran (THF). The mixture was cooled to -10°C, and then titanium tetrachloride (3.3 mL, 30 mmol) and pyridine (0.15 mL, 1.8 mmol) were added dropwise. The reaction mixture was refluxed at 85°C for 2 hours and then cooled to 0°C. A solution of di-p-tolyl ketone (3.15 g, 15 mmol) in 60 mL of dry THF was added. The resulting mixture was stirred at room temperature for 10 minutes before refluxing at 85°C for 12 hours. After the reaction was complete, the solution was cooled to room temperature and poured into 120 mL of 40% aqueous potassium carbonate solution. The mixture was stirred vigorously for 5 minutes, filtered, and the filtrate was extracted with dichloromethane. The organic phase was washed with water and saturated saline, dried over Na_2SO_4, and concentrated under reduced pressure to yield a yellowish solid. The crude product was purified by column chromatography (silica gel, petroleum ether) to afford 2.36 g of a white solid product in 75% yield. ^1H NMR (400 MHz, $CDCl_3$): δ 6.89 (s, 16H), 2.26 (s, 12H).

1,1,2,2-tetra-kis(4-(bromomethyl)phenyl)-ethene (TET): To a 100 mL Schlenk vial under an argon atmosphere, 0.78 g (2 mmol) of 1,1,2,2-tetra-p-tolyl ethylene obtained from the previous step was added, followed by NBS (1.71 g, 9.6 mmol), AIBN (0.06 g), and benzene (30 mL). The mixture was heated at 85°C for 24 hours, then cooled to room temperature and filtered. The clarified filtrate was concentrated under reduced pressure to yield a yellow solid. The crude product was purified by column chromatography (silica gel, petroleum ether/dichloromethane, 3:1 v/v) to afford 1.2 g of a light yellow solid product in 85% yield. ^{1}H NMR (400 MHz, $CDCl_3$): δ 7.13 (m, 8H), 6.97 (d, 8H), 4.42 (s, 8H).

Synthesis of BPB.

Synthesis of 1,4-bis(4-pyridyl)benzene (BPB) refers to previous literature (**Figure S1**) [2]. Under an argon atmosphere, 1,4-dibromobenzene (4.0 g, 17 mmol), 4-pyridinylboronic acid (6.3 g, 51.1 mmol), tetrakis(triphenylphosphine)palladium (0.98 g, 0.85 mmol), and sodium hydroxide (2.7 g, 68.1 mmol) were added to a 250 mL Schlenk vial. The vial was filled with a toluene/ethanol/water solution (250 mL, 3:2:1 v/v), and then placed in an oil bath at 120°C with constant stirring for 48 hours. After cooling to room temperature, the solvent was removed under reduced pressure. The solid residue was extracted with dichloromethane, washed with water and saturated aqueous saline solution, dried over anhydrous Na_2SO_4, and concentrated under reduced pressure to yield the crude product. The crude product was purified by column chromatography (silica gel, dichloromethane/ethyl acetate, 1:3 v/v) to afford 2.64 g of a white solid product in 67% yield. ^{1}H NMR (400 MHz, $CDCl_3$): δ 8.71 (s, 4H), 7.78 (s, 4H), 7.57 (s, 4H).

Synthesis of DBP.

Synthesis of 4,4’-(2,5-dimethoxy-1,4-phenylene)bis[pyridine] (DBP) refers to previous literature (**Figure S1**) [3]. To a 250 mL Schlenk flask under an argon atmosphere, 1,4-dibromo-2,5-dimethoxybenzene (2.16 g, 7.30 mmol), 4-pyridine boronic acid (1.91 g, 15.5 mmol), tetrakis(triphenylphosphine)palladium (856 mg, 0.741 mmol), and cesium carbonate (6.04 g, 18.5 mmol) were sequentially added. The flask was then filled with 120 mL of a 1:1 volume ratio of 1,4-dioxane and water solution, and the mixture was degassed by bubbling argon for

30 minutes. The reaction was carried out at 90°C for 24 hours. After cooling to room temperature, the solvent was removed under reduced pressure. The solid residue was extracted with dichloromethane, washed with water and saturated aqueous saline solution, and dried over anhydrous Na_2SO_4. The organic phase was concentrated under reduced pressure to yield the crude product. The crude product was purified by column chromatography (silica gel, dichloromethane/ethyl acetate, 1:1 v/v) to afford 1.2 g of a light yellow solid product in 56% yield. ^1H NMR (400 MHz, $CDCl_3$): δ 8.66 (d, 4H), 7.51 (d, 4H), 7.00 (s, 2H), 3.83 (s, 6H).

Synthesis of three ionic porous organic polymers.

In each case, TET (176 mg) and BIP (80 mg), BPB (116 mg), or DBP (146 mg) were sequentially added to a 25 mL Schlenk vial, followed by the addition of 5 mL of dried NMP under an argon atmosphere. The mixture underwent degassing through three freeze-thaw cycles and was subsequently stirred at 110 °C in an oil bath for 7 days. Upon completion, the reaction was allowed to cool to room temperature, filtered, and sequentially washed with dichloromethane, DMF, water, acetone, tetrahydrofuran, and methanol. Finally, the crude product was Soxhlet extracted with tetrahydrofuran and methanol for 48 hours, then dried under vacuum at 110 °C overnight to yield TET-BIP (yield 70%), TET-BPB (yield 79%), and TET-DBP (yield 81%) as reddish-brown, yellow, and orange-red solid powders, respectively.

Adsorption experiments of anionic dyes

To obtain the adsorption kinetic data, two anionic dye solutions Methyl Orange (MO) and Congo Red (CR) were prepared by dissolving the appropriate amounts of the dyes in deionized water. Then, 2 mg of polymer was added to 5 mL of 300 mg/L MO solution and 2 mg of polymer to 5 mL of 400 mg/L CR solution for the adsorption kinetic studies. The samples were stirred at room temperature, and the suspension was filtered through a membrane. The adsorbed dye concentration was measured at various intervals using a UV-visible spectrophotometer to determine the absorbance of the solution. The adsorption capacity (q_t) at time t was determined using the following Equation (S1):

$$q_t = \frac{(C_0 - C_t)V}{m} \quad \text{(S1)}$$

Here, C_0 and C_t represent the initial dye concentration and the concentration at time t (mg/L),

respectively, V denotes the solution volume (mL), and m refers to the mass of the adsorbent (mg).

To acquire adsorption isotherm data, 2 mg of the polymer was introduced into 5 mL of MO solution with initial concentrations of 500, 600, 700, 800, and 900 mg/L, and 0.5 mg of the polymer was added to 5 mL of CR solution with initial concentrations of 200, 400, 600, 800, and 1000 mg/L, respectively. Following a 24-hour stirring period at room temperature, the mixture was filtered through a membrane, and the absorbance of the solution was measured using a UV-visible spectrophotometer. The adsorption capacity (q_e) at equilibrium was calculated using the following Equation (S2):

$$q_e = \frac{(C_0 - C_e)V}{m} \tag{S2}$$

Here C_0 and C_e represent the initial and equilibrium concentrations (mg/L), V denotes the solution volume (mL), while m indicates the mass of the adsorbent (mg).

The polymer was isolated through centrifugation and subsequently washed with ethanol, methanol, and acetone to conduct desorption studies. The concentration of the supernatant was quantified using a UV-Vis spectrophotometer. The adsorbent was then employed again in a subsequent adsorption-desorption cycle. The reusability of the polymer was assessed by repeating the adsorption-desorption cycle five times, following the previously described procedure.

Adsorption experiments of Cr (VI)

A potassium dichromate solution was prepared by dissolving a precise quantity of potassium dichromate in deionized water. The kinetics of adsorption were investigated by introducing 2 mg of polymer into 5 mL of a 150 mg/L potassium dichromate solution. The samples were agitated at room temperature, and the resulting suspension was subsequently filtered through a membrane. The concentration of the adsorbed dye was quantified at various time intervals using a UV-visible spectrophotometer to assess the absorbance of the solution. The adsorption capacity (q_t) at time t was calculated by Equation (S1).

To determine the adsorption isotherms, 2 mg of the polymer was introduced into 5 mL of potassium dichromate solution at initial concentrations of 100, 200, 300, 400, and 600 mg/L. Following a 24-hour stirring period at room temperature, the mixtures were filtered through a

membrane, and the absorbance of the resulting solutions was measured using a UV-visible spectrophotometer. The adsorption capacity (q_e) at equilibrium was calculated by Equation (S2).

In the desorption studies, the polymers were separated by centrifugation and ion-exchanged by stirring overnight in a saturated potassium bromide solution, then washed with methanol and ethanol. Using a UV-visible spectrophotometer, the supernatant concentration was determined, and the adsorbent was subsequently reused for the next adsorption-desorption cycle. To evaluate the polymer's durability, the adsorption-desorption process was performed five times by the described procedure.

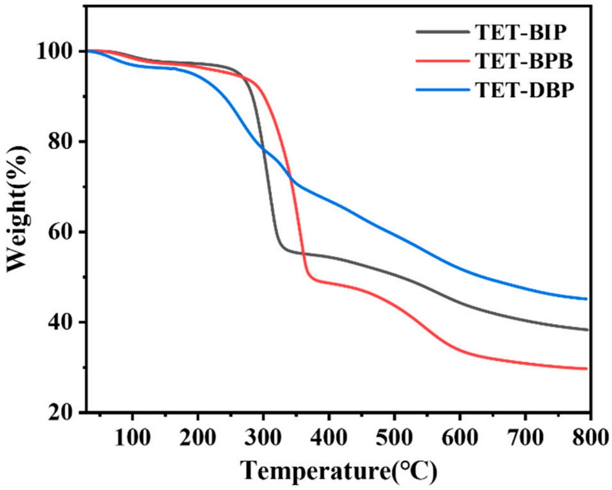

Figure S2. Thermogravimetric curves of TET-BIP, TET-BPB and TET-DBP.

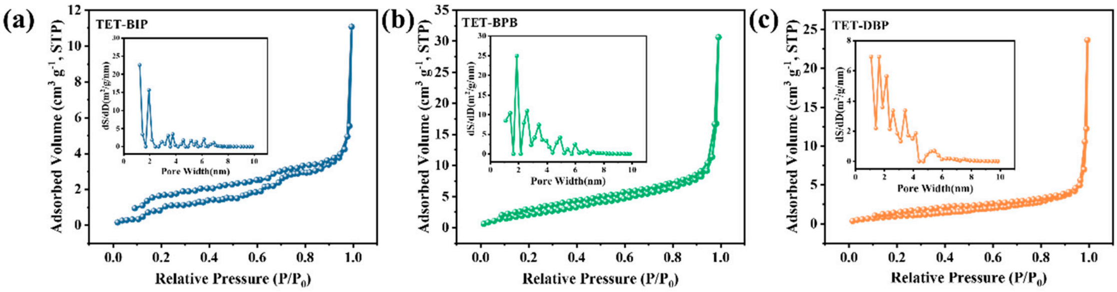

Figure S3. N_2 adsorption-desorption isotherms of (a)TET-BIP, (b)TET-BPB, and (c)TET-DBP (inset: pore size distributions).

Table S1. The BET surface area and porosity parameters of TET-BIP, TET-BPB, and TET-DBP.

Sample	S_{BET} (m^2 g^{-1})	Pore size(nm)	Pore Volume (cm^3 g^{-1})
**TET-BIP**	5.13	1.91	0.014
**TET-BPB**	9.32	2.10	0.045
**TET-DBP**	6.94	2.10	0.021

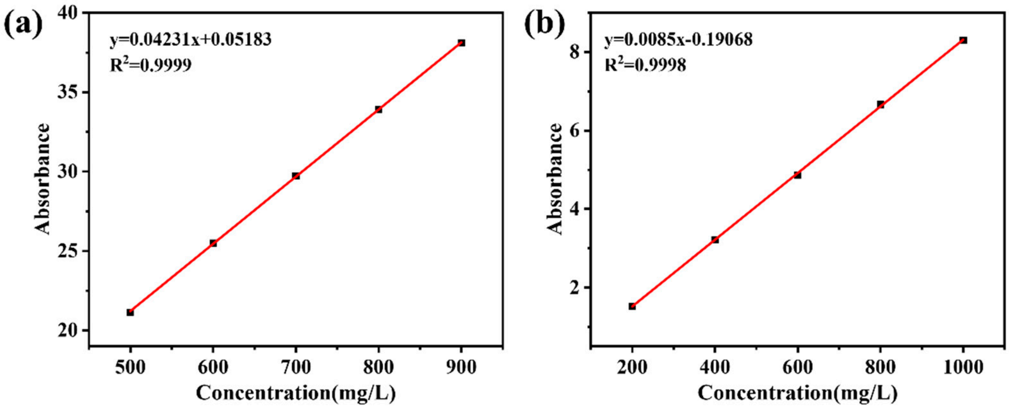

Figure S4. (a) Standard curve for MO, (b) Standard curve for CR.

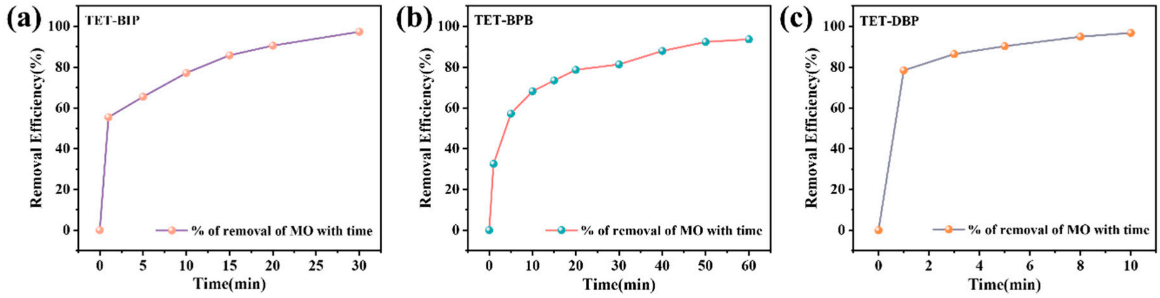

Figure S5. (a) TET-BIP, (b) TET-BPB, and (c) TET-DBP for MO removal efficiency over time.

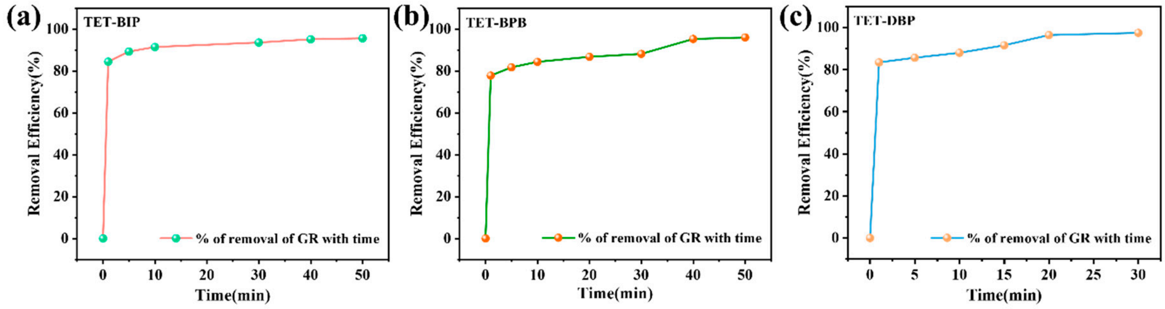

Figure S6. (a) TET-BIP, (b) TET-BPB, and (c) TET-DBP for CR removal efficiency over time.

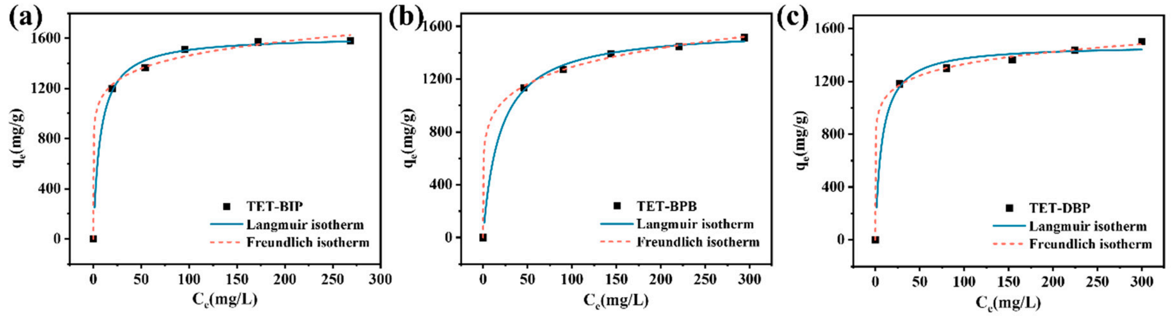

Figure S7. Langmuir and Freundlich model fitting of (a) TET-BIP, (b) TET-BPB, and (c) TET-DBP for MO.

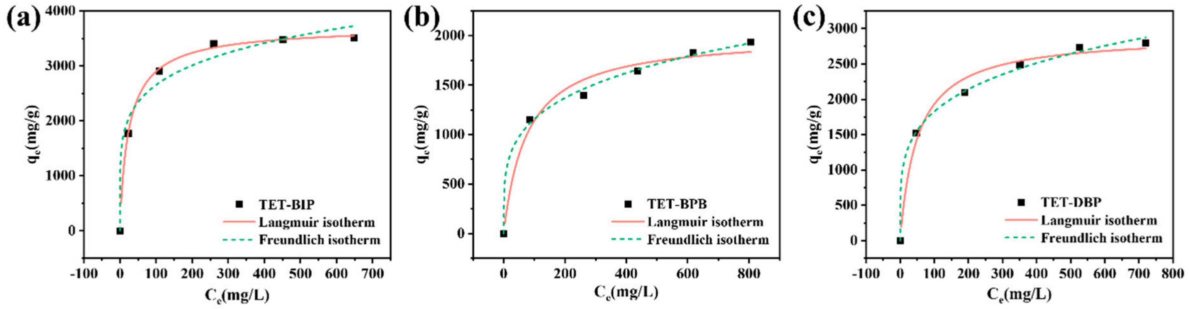

Figure S8. Langmuir and Freundlich model fitting of (a) TET-BIP, (b) TET-BPB, and (c) TET-DBP for CR.

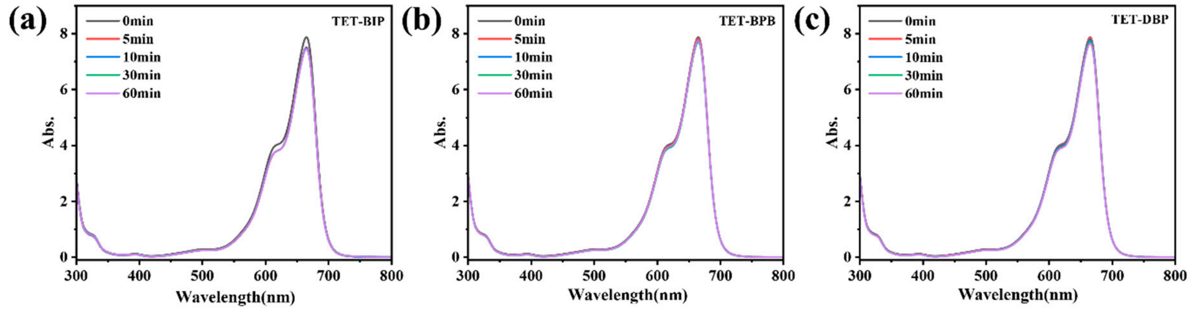

Figure S9. The changes of UV–Vis spectra of MB solution with the addition of (a) TET-BIP (b) TET-BPB and (c) TET-DBP at different time intervals.

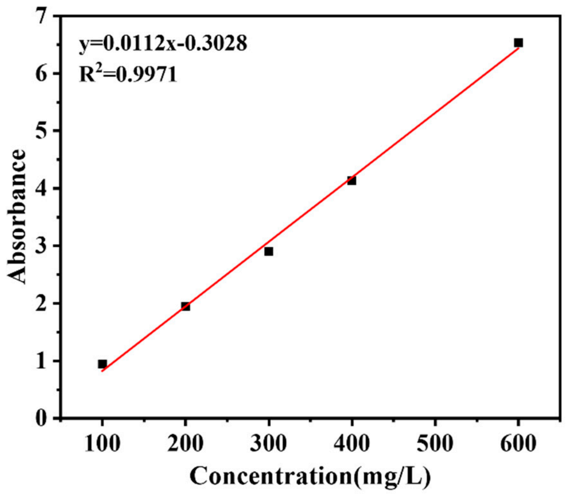

Figure S10. Standard curve for $Cr_2O_7^{2-}$.

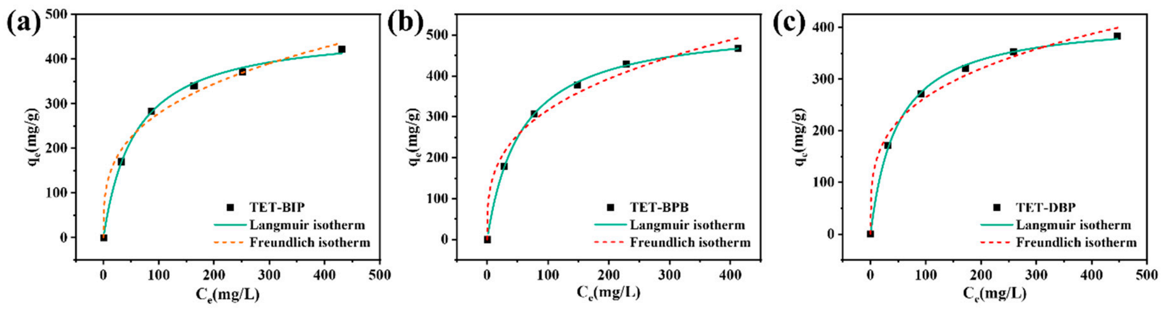

Figure S11. Langmuir and Freundlich model fitting of (a) TET-BIP, (b) TET-BPB, and (c) TET-DBP for $Cr_2O_7^{2-}$.

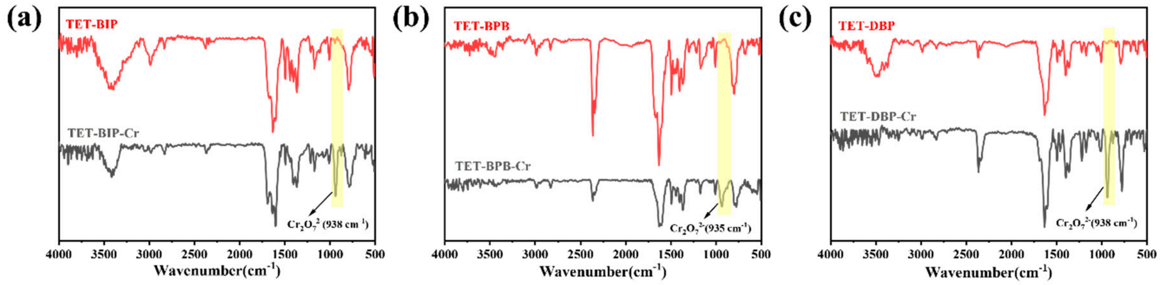

Figure S12. FTIR spectra of (a) TET-BIP, (b) TET-BPB, and (c) TET-DBP before and after adsorption of $Cr_2O_7^{2-}$.

References

1. Zhu, Y.-X.; Wei, Z.-W.; Pan, M.; Wang, H.-P.; Zhang, J.-Y.; Su, C.-Y. A New TPE-Based Tetrapodal Ligand and Its Ln(III) Complexes: Multi-Stimuli Responsive AIE (Aggregation-Induced Emission)/ILCT(Intraligand Charge Transfer)-Bifunctional Photoluminescence and NIR Emission Sensitization. *Dalton Trans.* **2016**, *45* (3), 943–950. https://doi.org/10.1039/C5DT03640B.
2. Scott, H. S.; Shivanna, M.; Bajpai, A.; Madden, D. G.; Chen, K.-J.; Pham, T.; Forrest, K. A.; Hogan, A.; Space, B.; Perry Iv, J. J.; Zaworotko, M. J. Highly Selective Separation of C2 H2 from CO2 by a New Dichromate-Based Hybrid Ultramicroporous Material. *ACS Appl. Mater. Interfaces* **2017**, *9* (39), 33395–33400. https://doi.org/10.1021/acsami.6b15250.
3. Feng, Y.; Das, P. J.; Young, R. M.; Brown, P. J.; Hornick, J. E.; Weber, J. A.; Seale, J. S. W.; Stern, C. L.; Wasielewski, M. R.; Stoddart, J. F. Alkoxy-Substituted Quadrupolar Fluorescent Dyes. *J. Am. Chem. Soc.* **2022**, *144* (37), 16841–16854. https://doi.org/10.1021/jacs.2c04906.
